# You are what you eat—ecological niche and microhabitat influence venom activity and composition in aquatic bugs

**DOI:** 10.1098/rspb.2022.2064

**Published:** 2023-03-29

**Authors:** Maike L. Fischer, Sol A. Yepes Vivas, Natalie Wielsch, Roy Kirsch, Andreas Vilcinskas, Heiko Vogel

**Affiliations:** ^1^ Department of Insect Symbiosis, Max-Planck-Institute for Chemical Ecology, 07745 Jena, Thüringen, Germany; ^2^ Department of Biochemistry, Max-Planck-Institute for Chemical Ecology, 07745 Jena, Thüringen, Germany; ^3^ Research Group Mass Spectrometry/Proteomics, Max-Planck-Institute for Chemical Ecology, 07745 Jena, Thüringen, Germany; ^4^ Institute for Insect Biotechnology, Justus Liebig Universitat Giessen, 35392 Giessen, Hessen, Germany

**Keywords:** ecological niche, diet, feeding style, venom, water bugs, proteotranscriptomics

## Abstract

True water bugs (Nepomorpha) are mostly predacious insects that live in aquatic habitats. They use their piercing–sucking mouthparts to inject venomous saliva that facilitates the capture and extra-oral digestion of prey animals, but their venom can also be deployed for defence. In Central Europe, nepomorph species representing different families coexist in the same habitat. However, their feeding ecology, including venom composition and deployment, has not been investigated in detail. We used an integrated proteotranscriptomic and bioactivity-based approach to test whether venom composition and activity differ between four water bug species sharing the same habitat but occupying different ecological niches. We found considerable species-dependent differences in the composition of digestive enzymes and venom components that probably evolved as adaptations to particular food sources, foraging strategies and/or microhabitats. The venom of *Corixa punctata* differed substantially from that of the three strictly predatory species (*Ilyocoris cimicoides*, *Notonecta glauca* and *Nepa cinerea*), and the abundance of herbivory-associated proteins confirms a mostly plant-based diet. Our findings reveal independent adaptations of the digestive and defensive enzyme repertoires accompanied by the evolution of distinct feeding strategies in aquatic bugs.

## 1. Introduction

True bugs (Heteroptera) are a diverse group of hemimetabolous insects that exploit a wide range of habitats and food sources around the world [1]. Although most true bugs feed on plants, recent phylogenetic studies suggest that heteropterans shifted to a predatory lifestyle when they diverged from the remaining phytophagous Hemiptera [2]. The infraorder Nepomorpha (true water bugs) comprises 11–13 families of almost exclusively predacious species that spend most of their lives under water [1]. Specific adaptations to their predatory lifestyle include the evolution of a strong but short rostrum [1], raptorial legs [3,4] and the secretion of venomous saliva that is used to paralyze, kill and pre-digest animal prey, but also in defence against enemies [1,5–8]. Some water bug venoms have remarkable effects on animals, including changes in contractile force and coronary flow in guinea pigs [9], paralysis in fish [10] and systolic arrest in cockroach heart–dorsum preparations [11].

The salivary/venom glands of true bugs usually feature three spatially separated parts: an anterior main gland (AMG), a posterior main gland (PMG) and an accessory gland (AG) [12]. The context-dependent deployment of AMG and PMG venom has been proven only for the terrestrial predacious assassin bugs *Pristhesancus plagipennis* and *Psytalla horrida* [7,8]. Differential venom deployment is not known among water bugs, although the analysis of AMG and PMG venom from the giant water bug *Lethocerus distinctifemur* (Nepomorpha, Belostomatidae) showed that the glands secrete distinct sets of proteins, suggesting different functional roles [13]. Proteins identified in water bug venoms include proteases, haemolysin-like proteins, protease inhibitors, hyaluronidases, phospholipases, amylases and numerous uncharacterized peptides [10,13–15]. However, most studies thus far have focused on belostomatid venoms so the dynamics of venom composition across different nepomorph families remain unclear, particularly when considering adaptation to microhabitats, differences in prey (or even shifts to a non-predacious lifestyle) and changes in predatory selection pressure.

**Figure 1.**
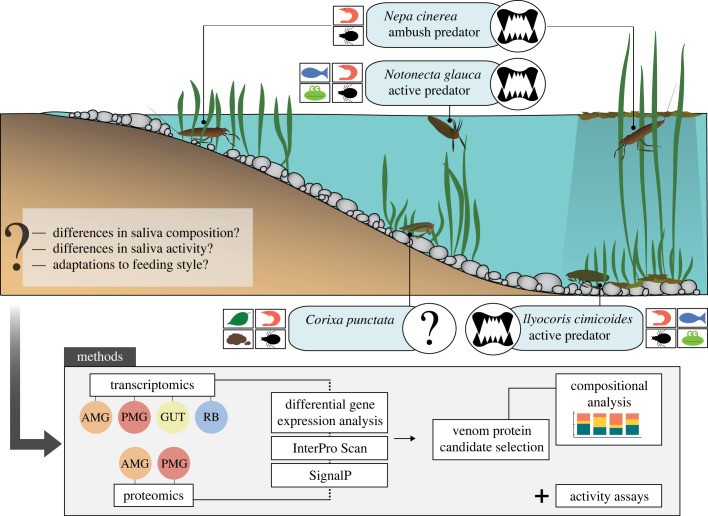
Schematic overview of the microhabitat preferences, food spectra (insects, crustaceans, fish, tadpoles, plants and detritus), feeding styles and foraging strategies of the water bugs *I. cimicoides, N. glauca*, *N. cinerea* and *C. punctata*, based on our experimental observations and the literature. We applied a proteotranscriptomic and bioassay-based approach to identify salivary proteins and saliva activity. AMG, anterior main gland; GUT, gut tissue; PMG, posterior main gland; RB, remaining body tissue.

Common European water bugs include the saucer bug *Ilyocoris cimicoides* (Naucoridae), the backswimmer *Notonecta glauca* (Notonectidae), the water scorpion *Nepa cinerea* (Nepidae) and the lesser water boatman *Corixa punctata* (Corixidae). These species coexist in the same type of habitat but differ in terms of microhabitat preferences, food spectra and foraging strategies (figure 1). *Ilyocoris cimicoides*, *N. glauca* and *N. cinerea* are strict predators of insects [16–20], crustaceans [16,21–23], tadpoles [24,25] and fish [26–28], but the dietary habits of *C. punctata* are not yet fully understood. Different studies have reported inconsistent feeding styles for *C. punctata*, ranging from strict zoophagy [29] to saprophagy [30] and omnivorous behaviour, including the unusual ability to ingest solid food [31]. Furthermore, foraging strategies differ among the predatory species. *Nepa cinerea* is a slow-moving ambush predator that hides in vegetation close to the water surface and quickly grabs approaching prey using its highly specialized predatory forelegs [3,20,29,32]. By contrast, *N. glauca* and *I. cimicoides* are good swimmers and actively hunt their prey [16,32,33]. *Notonecta glauca* usually searches for prey on the water surface or swimming in the open water [18,34,35], whereas *I. cimicoides* remains on the sediment or in vegetation to hunt and feed on prey [32,33]. These microhabitat preferences may be associated with different food spectra given that the composition of the prey community differs between microhabitats [34].

The effects of ecological niches on saliva composition and activity in heteropterans are poorly understood and little is known about the consistency of adaptations to specific conditions. Previous studies have focused on terrestrial bugs and mainly investigated differences in the activity of digestive enzymes to draw conclusions about dietary habits [36–39]. Recently, the salivary protein composition was analysed in several terrestrial and two aquatic bugs from different suborders, revealing patterns that may indicate specific dietary habits [40]. The proteins characteristic of predatory bugs included CUB domain proteins, haemolysins, Ptu1-like peptides, redulysins and several uncharacterized peptides. By contrast, typical herbivore-associated proteins included amylases, glucosidases, vitellogenins and cathepsins. However, few studies have considered species in the same infraorder with different ecological adaptations. We hypothesized that aquatic bug species sharing the same habitat but occupying different ecological niches (including microhabitat, food spectrum and foraging strategy) would have different venom compositions and activities. Our main objectives were, therefore, to collect and analyse gland-specific gene expression data and venom gland protein compositions using an integrated proteotranscriptomic and bioassay-based approach, followed by the comparison of venom composition and activity in *I. cimicoides*, *N. glauca*, *N. cinerea* and *C. punctata* to identify interspecific differences and relationships with each ecological niche. We discuss the ecological insights that can be drawn from these results in order to determine whether it is possible to infer the ecology of a given heteropteran species from the composition of its venom, or *vice versa* (figure 1).

## 2. Material and methods

### (a) Insects and venom collection

Specimens of *I. cimicoides*, *N. glauca*, *N. cinerea* and *C. punctata* were collected in Thuringia, Germany and kept in water-filled containers before dissection. Venom likely to have a defensive function was obtained by harassment. Specimens were captured with forceps and held above the water, which elicited defensive behaviour. An artificial prey dummy was built by enclosing a droplet of phosphate-buffered saline (PBS) within a piece of stretched Parafilm (electronic supplementary material, figure S1) and was offered to the insects for venom injection [7]. The artificial prey dummy was moved on the water surface to simulate moving prey and induce hunting behaviour. When a bug attacked the artificial prey, it was allowed to inject saliva for 1.5 min before removing the dummy and recovering the venom. It was not possible to collect defensive venom from *N. cinerea* or defensive and prey-killing venom from *C. punctata*. In addition to non-invasive collection, venom was also extracted directly from the venom glands of fifth-instar or adult bugs. The specimens were anaesthetized at –20°C for 5 min before dissection in PBS. The AG, AMG and PMG (only AMG and PMG for *C. punctata*) were separated and placed in pre-cooled tubes containing 10–20 µl PBS on ice. The samples were briefly vortexed and centrifuged (4000 g, 3.5 min) and the supernatant was transferred to a fresh tube. The venom of several individuals was pooled and stored at –20°C for analysis. The total protein concentration in the samples was measured using an N60 nanophotometer (Implen).

### (b) Proteomic analysis

The proteins in the venom samples were separated by sodium dodecylsulfate–polyacrylamide gel electrophoresis (SDS–PAGE) using 4–12% Criterion XT gradient gels (Bio-Rad) with XT MES running buffer at 125 V for 1.5 h, alongside protein molecular weight markers. The gels were stained for 1.5 h using a 1 : 1 mixture of Coomassie Brilliant Blue R-250 and colloidal Coomassie Brilliant Blue G-250 (Thermo Fisher Scientific), washed in Millipore water overnight and then scanned. For liquid chromatography with tandem mass spectrometry (LC–MS/MS) analysis, protein bands from each gel lane were excised and digested with trypsin [41]. Further details of LC–MS sample processing, data acquisition and data processing are presented in electronic supplementary material, Methods S1, section 1.

### (c) Venom gland collection and RNA isolation

AMG, PMG, gut and remaining body tissue (fat body, muscle tissue and integument) were carefully removed and placed in separate ceramic bead tubes containing 500 µl TRI Reagent (Sigma-Aldrich). The tissues of several individuals were pooled and homogenized using a TissueLyser LT (Qiagen). Total RNA was extracted using the Direct-zol RNA Miniprep Kit (Zymo Research). The quantity of RNA was measured using the Implen N60 nanophotometer and RNA integrity was confirmed using an Agilent 2100 Bioanalyzer and RNA Nanochip (Agilent Technologies).

### (d) RNA-Seq and *de novo* transcriptome assembly

For all species, transcriptome sequencing of AMG, PMG, gut and remaining body tissue was performed by the Max-Planck Genome Center Cologne (http://mpgc.mpipz.mpg.de/home/) using an Illumina HiSeq3000 Genome Analyzer platform. Poly(A) mRNA was extracted from 1 µg total RNA using oligo-dT attached to magnetic beads, and was fragmented to an average length of 250 bp. Sequencing libraries were generated using the TruSeq RNA library preparation kit (Illumina) and paired-end (2 × 150 bp) read technology was used for sequencing. All generated reads were processed using an in-house assembly and annotation pipeline as previously described [42]. Details of assemblies, annotations and RNA-Seq mapping are presented in electronic supplementary material, Methods S1, section 2.

### (e) Venom activity bioassays

**Figure 2.**
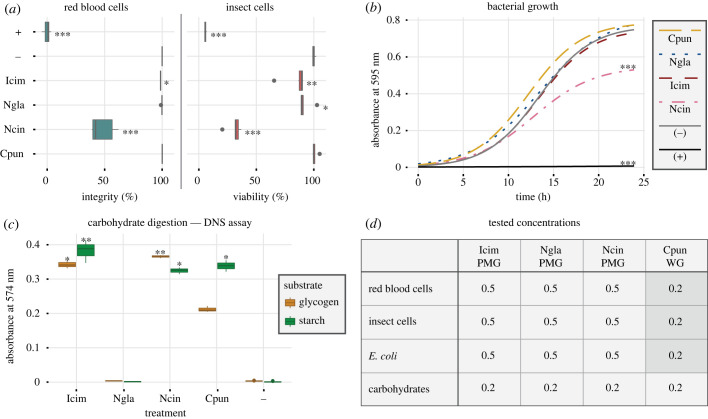
Effects of PMG extracts or whole gland (WG = AMG + PMG) extracts on different substrates. Significant differences compared to the negative control are highlighted with asterisks (*p ≤ 0.05; **p ≤ 0.01; ***p ≤ 0.001). Box plots present the median (line), interquartile range (box) and data range (whiskers). (*a*) Effects on horse erythrocyte integrity and insect cell viability: (−) = PBS; (+) = 0.1% Triton X-100. Statistical analysis: Dunn's test, *n* = 3 (haemolysis), *n* = 6 (cell viability). Icim, *I. cimicoides;* Ngla, *N. glauca*; Ncin, *N. cinerea*; Cpun, *C. punctata.* (*b*) Effects on *E. coli* growth: (−) = PBS; (+) = 0.05 mg ml^−1^ gentamycin. The data were fitted to a logistic model and plotted as growth curves. Statistical analysis: permutation test, *n* = 3. (*c*) Digestion of amylose and glycogen: (−) = PBS. Statistical analysis: Dunn's test, *n* = 3. (*d*) Summary of the final venom concentrations (mg ml^−1^) tested in the different bioassays.

Haemolytic activity was determined on defibrinated horse blood (Thermo Fisher Scientific). Erythrocytes were harvested by centrifugation (1500 g, 3 min), washed three times with PBS and prepared as a 1 : 10 erythrocyte suspension in PBS. We mixed 20 µl venom extract in PBS (concentrations shown in figure 2) with 180 µl of the cell suspension (*n* = 3) in a 96-well plate and incubated the cells at 37°C for 1 h. We used 1% Triton X-100 and PBS as positive and negative controls, respectively (*n* = 3). The cells were centrifuged (2000 g, 10 min) and the supernatants were transferred to a clear 96-well plate. The absorbance at 440 nm was measured using an Infinite m200 plate reader (Tecan). Relative haemolysis was calculated in relation to the positive control (defined as 100%).

Bacterial growth inhibition was tested using a liquid growth antibacterial assay with *Escherichia coli*. We inoculated 50 µl of an *E. coli* overnight culture into 5 ml lysogeny broth (LB) and incubated the cells at 37°C for 2–3 h. The culture was diluted with LB to an optical density at 600 nm (OD_600_) of 0.003 and 90 µl of the bacterial suspension were mixed with 10 µl venom extract in PBS (concentrations shown in figure 2) in a clear, sterile 96-well plate. We used 0.05 mg ml^−1^ gentamycin and PBS as the positive and negative controls, respectively. The absorbance at 595 nm was measured using the Tecan Infinite m200 plate reader over a period of 24 h at 5 min intervals. The temperature was held constant at 30°C. Relative growth inhibition was calculated in relation to the positive control (defined as 100%) at the time the growth control reached OD_595_ = 0.35.

Cytotoxic effects against *Spodoptera frugiperda* (Sf9) cells were tested using an MTT assay based on thiazolyl blue tetrazolium bromide. The cells were cultured in Sf-900 II SFM medium (Gibco) containing 0.05 mg ml^−1^ gentamycin in a sterile 96-well plate (Thermo Fisher Scientific). After 24 h, the culture medium was replaced with 100 µl venom extract in culture medium (concentrations shown in figure 2). We used 100 µl 0.1% Triton X-100 and 100 µl culture medium as positive and negative controls, respectively. The cells were incubated at 27°C for 4 h. The culture medium was then replaced with 100 µl 0.5 mg ml^−1^ MTT solution in culture medium and incubated at 27°C for 2 h. The MTT solution was removed and replaced with 50 µl DMSO per well. After incubation at 27°C for 10 min, the plate was briefly vortexed, and the absorbance at 540 nm was measured using the Tecan Infinite m200 plate reader. The relative cell viability was calculated in relation to the negative control (defined as 100%).

### (f) Carbohydrase activity assay

The degradation of starch and glycogen by venom extracts was measured using the 3,5-dinitrosalicylic acid (DNS) method as previously described [43]. Briefly, crude venom extracts in PBS (concentrations shown in figure 2) were mixed with either 1% (w/v) starch or 1% (w/v) glycogen in water at a ratio of 3 : 1 (v/v) and incubated at 25°C for 2 h. An equal volume of a 99 : 1 (v/v) mixture of solution 1 (44 mM DNS, 21 mM phenol, 250 mM sodium hydroxide) and solution 2 (400 mM sodium sulfide) was added to each reaction and incubated at 99°C for 5 min. We then added 200 mM potassium sodium tartrate at a ratio of 1 : 6 (v/v) and measured the absorbance at 575 nm using the Tecan Infinite m200 plate reader.

### (g) Statistical analysis

Statistical analysis was conducted using R v.4.0.3 and the integrated development environment RStudio v.1.2.1335 (http://www.R-project.org/). For the haemolysis, cytotoxicity and carbohydrase activity assays, we performed Kruskal–Wallis tests with subsequent Dunn's test for multiple comparisons using the FSA package [44] in order to identify significant differences compared to the negative controls. For the analysis of bacterial growth curves, the data were fitted to a logistic model using the growthcurver package [45]. Permutation tests for pairwise comparisons of growth curves were performed using the statmod package [46].

## 3. Results

### (a) Venom activity toward different substrates

We carried out bioactivity assays on different cells and substrates to identify species-dependent differences in venom activity. The *N. cinerea* PMG extract showed strong toxicity toward horse erythrocytes and Sf9 cells, and inhibited the growth of *E. coli*. By contrast, the *I. cimicoides* and *N. glauca* PMG extracts showed little and no activity, respectively, against erythrocytes, and only mild toxicity toward Sf9 cells. Whole gland extracts (AMG + PMG) from *C. punctata* showed no toxicity toward any of the cells (figure 2*a*,*b*). The ability of extracts to degrade the polysaccharides starch and glycogen was determined using the DNS method by quantifying the reducing groups released during substrate hydrolysis. The extracts from *I. cimicoides*, *N. cinerea* and *C. punctata* were able to degrade both substrates, whereas the PMG extract from *N. glauca* did not digest either of them (figure 2*c*).

### (b) Glandular origin of defence and predation venom

**Figure 3.**
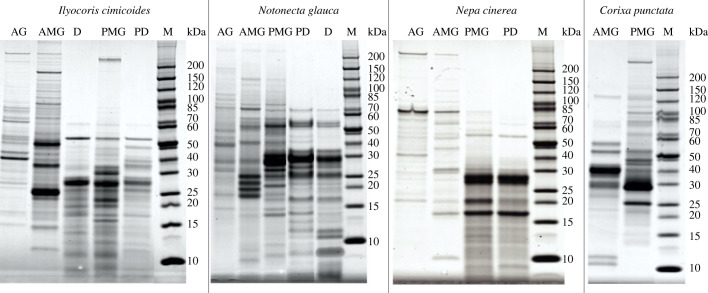
SDS–PAGE analysis of venom gland extracts and venom collected non-invasively from *I. cimicoides*, *N. glauca*, *N. cinerea* and *C. punctata*. AG, accessory gland extract; AMG, anterior main gland extract; PMG, posterior main gland extract; PD, prey dummy venom; D, defensive venom; M, protein marker (kDa).

**Figure 4.**
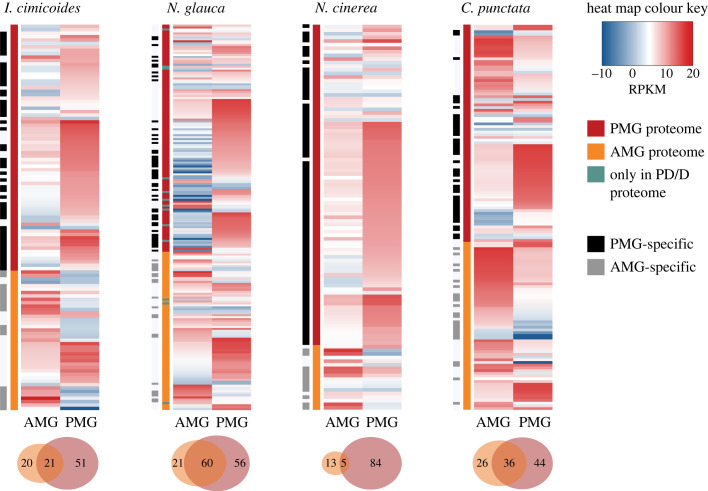
AMG-specific, PMG-specific and common gene expression in *I. cimicoides*, *N. glauca*, *N. cinerea* and *C. punctata*. The gene expression levels (RPKM) of the identified venom proteins in the AMG (highlighted in orange), PMG (highlighted in red) or only PD/D proteomes (highlighted in green) are shown in the heat maps. PMG-specific and AMG-specific proteins are shown using black and grey bars, respectively. The numbers of proteins unique to and common to the AMG and PMG in each species are visualized using Venn diagrams.

AG, AMG and PMG extracts, as well as non-invasively collected venom samples, were fractionated by SDS–PAGE to visually compare their banding patterns. The protein bands of prey dummy and defensive venoms resembled the PMG extracts of the corresponding species, suggesting that the PMG is the glandular origin of both venom types (figure 3). Proteomic analysis of excised bands showed that the AMG and PMG secrete distinct sets of proteins, but the proportion of gland-specific proteins differed between species. In *N. glauca*, 45% of the proteins were secreted by both glands, whereas in *N. cinerea* almost all proteins (95%) were specific to either the AMG or PMG (figure 4). Furthermore, most proteins in the prey dummy and defensive venoms were PMG-specific or produced by both lobes. Very few proteins were AMG-specific (electronic supplementary material, figure S2), confirming that both the prey dummy and defensive venoms originate from the PMG.

### (c) Protein composition of anterior main gland and posterior main gland venoms

**Figure 5.**
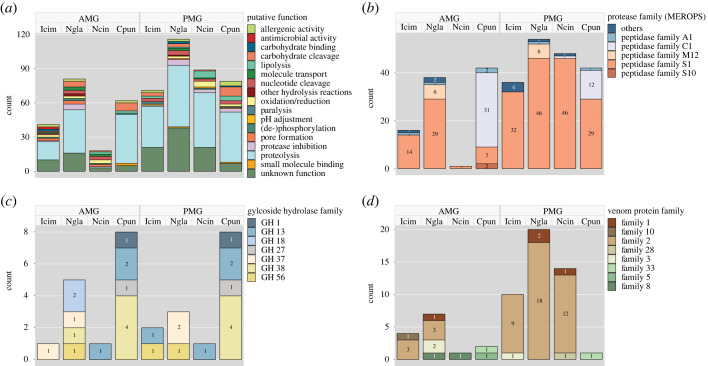
Protein composition of AMG/PMG venom in *I. cimicoides*, *N. glauca*, *N. cinerea* and *C. punctata* based on proteomics analysis and shown as relative transcript abundance. (*a*) Colour-coded blocks show the number of contigs identified by proteotranscriptomic analysis encoding specific classes of functional proteins. Categories are further subdivided into (*b*) protease families (MEROPS), (*c*) glycoside hydrolase families and (*d*) venom protein families.

Next-generation sequencing (RNA-Seq) was carried out to identify and quantify venom-associated transcripts, thus providing more insight into the protein composition of AMG and PMG venom. RNA isolated from the AMG, PMG, gut and remaining body tissue was used for Illumina sequencing, which yielded 30–40 million reads per sample. Information on the *de novo* reference transcriptome assemblies is provided in electronic supplementary material, table S1. Most of the identified proteins were proteases, followed by uncharacterized heteropteran venom proteins assigned to various families. Digestive enzymes such as lipases, carbohydrases, nucleases and nucleotidases were also detected (figure 5*a*). Tissue-specific RPKM levels showed that most venom proteins were associated with highly gland-specific gene expression patterns (figure 4). The most abundant transcripts in the PMG encoded S1 family peptidases and members of venom protein family 2 (as well as a CUB domain protein in *N. cinerea*), whereas the most abundant transcripts in the AMG encoded haemolysins, venom protein family 2 members and other uncharacterized proteins. In *C. punctata*, the most abundant AMG transcripts encoded C1 family peptidases. Ptu1-like peptides, which are common channel modulators in heteropteran venoms, were detected only in *I. cimicoides*. Remarkable interspecific differences were found among the digestive enzymes. In the three predatory species, most of the proteases were S1 family peptidases, whereas more than half of the proteases in *C. punctata* were C1 family peptidases, most of which were expressed in the AMG. Furthermore, several M12 family metallopeptidases were identified solely in *N. glauca* (figure 5*b*). In addition to proteases, several carbohydrases from different glycoside hydrolase (GH) families were also identified. The largest number of GHs was detected in *C. punctata* and included families GH1, GH13, GH27 and GH38. In the predatory species, we identified carbohydrases from families GH13, GH18, GH37, GH38 and GH56 (figure 5*c*). We also observed remarkable differences in the expression of uncharacterized heteropteran venom protein families. In *I. cimicoides*, *N. glauca* and *N. cinerea*, most such proteins belonged to venom protein family 2 and were strongly expressed in the PMG. By contrast, *C. punctata* expressed no venom protein family 2 proteins but did express two venom proteins from families 5 and 33, which were not present in the other species. We also identified venom proteins from families 1, 3, 8, 10 and 28 (figure 5*d*).

## 4. Discussion

We used an integrated proteotranscriptomic and bioactivity-based approach to investigate how ecological niches shape the venom/saliva protein composition and activity in four water bug species that coexist in the same aquatic habitats. The two main salivary glands (AMG and PMG) secreted distinct and complex sets of proteins, although only the PMG was found to be involved in envenomation. The composition and biological activity of the venoms differed between the four species, so we considered the impact of the microhabitat, food spectrum and foraging strategy in more detail.

Water bugs are well-adapted to an aquatic lifestyle and many different species coexist in the same habitat, but despite this the microhabitat preferences of individual species can differ considerably. The behaviour of each species reflects whether it spends its time primarily near the water surface, on vegetation, or on the sediment at the bottom of the pond, and this is likely to result in microhabitat-specific adaptations [17,35]. *Notonecta glauca* is an active predator, searching for prey on the water surface or in the water column, and it is, therefore, exposed to predators [16,18,34,35]. By contrast, *N. cinerea* usually hides in vegetation to wait for prey, but this low mobility may increase its susceptibility to predation [29,32]. Both species, therefore, rely on their potent venom to deter predators. Defensive venom generally induces pain, which motivates predators to quickly release their prey and also deters future attacks through avoidance behaviour [47–49]. For example, the haemolytic polypeptide melittin in bee venom induces intense pain in vertebrates [50,51], mainly by acting directly on primary nociceptive cells [52,53] but possibly also by disrupting mast cell membranes and causing tissue damage, thus triggering the release of pain-inducing compounds [53–55]. Pore-forming peptides from ants [56], spiders [57], fish [58] and bacteria [59] also have nociceptive effects on vertebrates. In our experiments, *N. cinerea* PMG venom had strong haemolytic, cytotoxic and antimicrobial effects, indicating the presence of lytic proteins that may be responsible for severe pain following envenomation [20]. By contrast, PMG venom from *N. glauca* did not show strong lytic activity, although the Notonectidae are known for their painful stings [60]. In snake venoms, metalloproteases induce potent hyperalgesia possibly by triggering mast cell activation [61–63]. Similarly, the role of metalloproteases in pain induction, myotoxicity and inflammation by centipede venom has been discussed [64,65]. We identified several M12 family metalloproteases solely in *N. glauca* venom, which suggests that such proteins are species-dependent adaptations to a microhabitat with higher risks of predation. However, a detailed characterization of *N. glauca* M12 family metalloproteases is necessary to clarify their function and putative role in pain induction.

Microhabitat preferences are also likely to affect feeding habits because the abundance and availability of food vary within a water body. *Notonecta glauca*, *I. cimicoides* and *N. cinerea* are generalist predators that feed on insects and crustaceans [16–19,21–23,29,30], and also on vertebrates such as fish and tadpoles in the case of *N. glauca* and *I. cimicoides* [24–28]. We identified a protein in *N. glauca* and *I. cimicoides* PMG venom that is homologous to venom 5′ nucleotidase 1 from the belastomatid *L. distinctifemur* [13]. Such enzymes are often found in snake, spider and true bug venoms and they inhibit platelet aggregation in vertebrate prey [13,66–69]. This indicates that *N. glauca* and *I. cimicoides* have adapted their venom composition for vertebrate prey, similarly to water bugs of the family Belastomatidae. Predatory species that feed on large, mobile animals require adaptations to quickly overwhelm their prey. This is facilitated by morphological structures such as raptorial forelegs or venom components that induce paralysis. A common channel modulator associated with paralytic activity in heteropteran venoms is Ptu1, an inhibitor cystine knot (ICK) family peptide first isolated from the assassin bug *Peirates turpis* [70,71]. Only *I. cimicoides* venom contained Ptu1-like peptides, and the transcripts were most abundant in the PMG. *Nepa cinerea* may not require paralytic venom because it uses its specialized predatory forelegs to prevent the escape of its prey [4]. However, the Notonectidae do not have well-developed raptorial forelegs and instead quickly paralyze their prey [72–74]. We found many uncharacterized proteins in *N. glauca* venom, including several peptides with no known homologues, suggesting that prey immobilization by this species is facilitated by other proteins with distinct mechanisms of action. One protein family that was particularly abundant and strongly expressed in the venom glands of the three predatory species was heteropteran venom protein family 2, a group also present in other zoophagous and haematophagous bugs from various families but not in the phytophagous species investigated thus far [7,8,13,40,75,76]. The role of these proteins is unclear, but their strict gland-specific expression and abundance in predatory species suggest a key role in predation. Their complete absence in *C. punctata* indicates a non-predatory lifestyle. Further research, including the heterologous expression and characterization of different venom protein family 2 members, is needed to determine their specific function in venom activity.

Unlike most water bugs, *C. punctata* reportedly feeds on detritus, algae, small insects and crustaceans [29–31]. In addition to this diet, its mouthpart morphology has also puzzled scientists because it differs considerably from the elongated rostrum typical of true bugs (electronic supplementary material, figure S3). These highly specialized mouthpart structures allow the Corixidae to ingest both liquid and solid food, a unique feature among heteropterans [31,74]. Mouthpart morphology and digestive enzymes play a key role in extra-oral digestion, and changes in enzyme composition may facilitate adaptation to different food sources. The ratio of amylase to protease activity has been used to predict heteropteran feeding habits, assuming that high protease and low amylase activity represent zoophagy, whereas low protease and high amylase activity represent phytophagy [39]. The structural similarity between starch and glycogen—the main carbohydrate storage products in plants and animals, respectively—may allow amylases to digest both substrates, as described for the midgut amylases of *Andralus spinidens* [77]. We found that *I. cimicoides*, *N. cinerea* and *C. punctata* venoms can digest both starch and glycogen, indicating that the presence of salivary amylases and amylase activity does not necessarily imply phytophagy. Predatory insects rely heavily on proteases to digest protein-rich animal prey. In true bugs, most salivary proteases are serine endopeptidases, which have optimal activity at basic or neutral pH [78–81]. By contrast, cysteine or aspartic endopeptidases (cathepsins) are typically found in gut secretions and are most active at acidic pH [78,79,81,82]. Surprisingly, most of the proteases detected in *C. punctata* saliva were cysteine-type C1 family peptidases. Salivary cysteine proteases have been found primarily in phytophagous hemipterans, where they digest plant-derived proteins and play a role in immunity against bacterial pathogens [83–86]. In addition, salivary cathepsins from aphids elicit plant defences during feeding [87]. The strong association between salivary cysteine proteases and herbivory suggests that *C. punctata* secretes C1 peptidase-rich saliva as an adaptation to a mainly plant-based diet. Similarly, the composition of polysaccharide-degrading GH families partially reflects feeding type differences between water bug species. Whereas some GH families (e.g. GH56 hyaluronidases, GH37 trehalases) are found exclusively in predacious species, targeting abundant polysaccharides in prey, bi-functional amylases (GH13) occur in predacious and herbivorous species. Predacious species could benefit from dual-function enzymes because the amylase/glycogenase GH13 enzyme could allow easier access to the major dietary polysaccharides, enabling (among other factors) potential host shifts from herbivory to carnivory.

Our study shows that the venom composition and activity differ among four aquatic bug species occupying different ecological niches within the same habitat. We found remarkable interspecific differences and identified venom proteins that probably facilitated adaptations to particular food sources, foraging strategies and/or microhabitats. Many different factors influence salivary composition and assumptions about the dietary habits of true bugs should not rely solely on compositional analysis. Furthermore, research covering a wide range of heteropteran infraorders and families, feeding habits and ecological niches is needed to derive general patterns and adaptations. Even so, it is clear that the compositional analysis of salivary venom provides strong evidence for the ecological adaptations of water bugs, and that the unique venom composition of *C. punctata*, including the abundance of herbivory-associated enzymes, is sufficient to hypothesize a predominantly plant-based diet in this species. Hypotheses based on venom protein profiles can, therefore, be used as the basis for additional experiments to determine the precise ecological niches occupied by hemipteran species.

## Data Availability

The data for this study have been deposited in the European Nucleotide Archive (ENA) at EMBL-EBI under accession number PRJEB58831 (https://www.ebi.ac.uk/ena/browser/view/PRJEB58831). The Illumina short read data can be found with the following sample accession numbers: ERS14412810-ERS14412813 (*Corixa punctata*), ERS14412814-ERS14412818 (*Ilyocoris cimicoides*), ERS14412819-ERS14412823 (*Nepa cinerea*) and ERS14412824-ERS14412827 (*Notonecta glauca*). The sequence and transcriptome assembly data have also been deposited in the Edmond Data Repository and are directly accessible via the following weblinks: https://doi.org/10.17617/3.VOQQRJ [88] (*Corixa punctata*), https://doi.org/10.17617/3.OSCGGN [89] (*Ilyocoris cimicoides*), https://doi.org/10.17617/3.7FEIDD [90] (*Nepa cinerea*) and https://doi.org/10.17617/3.B0WMNP [91] (*Notonecta glauca*). The data are provided in electronic supplementary material [92].
